# Editorial for the Special Issue on Lab-on-PCB Devices

**DOI:** 10.3390/mi13071001

**Published:** 2022-06-25

**Authors:** Francisco Perdigones

**Affiliations:** Electronic Devices and Systems Group (DISEL), Electronic Engineering Department, Higher Technical School of Engineering, University of Seville, 41092 Seville, Spain; fperdigones@us.es

**Keywords:** lab-on-PCB, printed circuit board, biomedical applications, electronics, sensors, biosensors, actuators, lab-on-chip, microfluidics

The use of Printed Circuit Boards (PCBs) has seen a remarkable growth over the last decade, with applications in engineering, medicine, biology, chemistry, etc. For example, many biosensors can be fabricated using Printed Circuit Board substrates, resulting in lab-on-PCB devices. In addition, these PCB-based biosensors have been integrated into microfluidic platforms to develop Lab-on-PCB systems [1]. The use of PCBs for developing lab-on-chip devices was proposed in 1996 [2], and the term “Lab-on-PCB” was coined in October 2014 [3]. Apart from sensors and biosensors, the lab-on-PCB devices can include actuators, such as microheaters [4], and devices for microfluid handling, for example, electrowetting on dielectric devices (EWOD) [5]. Since 1996, many lab-on-PCB devices have been developed. However, the majority of the contributions have been published during the last five years. These new contributions focus on the development of applications of current interest. Therefore, we can consider it as an emerging technology.

The lab-on-PCB devices are an interesting improvement of lab-on-chip devices due to the inexpensive characteristic of Printed Circuit Boards, and the possibility of including microfluidic circuits, sensors, and actuators, together with electronics in the same substrate. In addition, these characteristics lead the mass production of biomedical devices, and thus make these devices a very interesting choice from the market point of view.

This Special Issue comprises 10 original contributions related to several aspects of research and development of lab-on-PCB devices. It includes two review papers and eight regular research papers. One of these paper is related with the integration of new emerging rapid prototyping technique with lab-on-PCB devices. This prototyping technique is vat polymerization with an liquid-crystal-display (LCD) as mask, also named masked stereolithography (mSLA) [6]. The technique is available with good resolutions for microfluidics (down to 35 µm). The work reported in [7] describes a technology which creates microfluidics on a Printed Circuit Board substrate using a mSLA printer. The authors describe the steps of the production process, so that, the procedure can be carried out using commercially available printers and resins. This includes the structuring of the copper layer of the PCB and the fabrication of the microchannels layer on top of the Printed Circuit Board. In this respect, the authors fabricated a conductivity sensor chip where several electrode arrangements were demonstrated. They demonstrated, among others, the principle of salinity measurements using seawater and different electrode arrangements using the fabricated microfluidic chip. This technique is a very interesting choice for low cost and rapid prototyping microfluidic devices with integrated sensors.

An important aspect to develop integrated sensors and actuators on lab-on-PCB devices consists in studying the integration method of components. The study of the physical reliability of electronic components are important, principally for thermal processes. In this respect, the work reported in [8] performs a comparative thermal analysis of the cooling efficiency of a surface mounted device (SMD) resistor and an embedded component on a Printed Circuit Board substrate. The authors created a model of heat distribution on a PCB, which took into account the both conductive and convective heat exchange, and they confirmed the behavior with experiments. The results show, among others, that the temperature of the embedded component was less than the temperature of the SMD component under natural convection. These results could be taken into account when integrating sensors, actuators or electronics components on lab-on-PCB devices.

Another interesting issue related to PCB-based devices for biomedical applications is the study of the suitability of commercially available biocompatible materials. The work reported in [9] studied the biocompatibility of a commercial Printed Circuit Board for organotypic retina cultures. The authors cultured retinal explants over the white solder mask of a commercial company, on open and closed systems with promising results, that is, the cell viability data show that the white solder mask had no cytotoxic effect on the culture. These results mean a starting point for fabricating microelectrodes arrays (MEAs) on PCB, for regenerative medicine to develop inexpensive methods for improving vision.

Regarding the array of microelectrodes, the work reported in [10] shows a transition from a static MEA (no flow) to a dynamic MEA (continuous flow), assuring a homogeneous transfer of an electrolyte solution (KCl solution) across the measurement chamber. This microfluidic chamber is designed for ensuring both continuous and uniform flow of medium across the complete surface of the chip. The optimum design includes a rounded-corner microfluidic chamber with three inlets and three outlets following the electrode periphery. The results showed, among others, that the change in the voltage noise was minimal when changing from a static MEA to a dynamic one. This is specially interesting for developing long-term cell and tissue cultures using Printed Circuits Boards as substrate.

One of the most useful devices for biomedical applications are the biosensors, particularly electrochemical biosensors. Therefore, the achievement of a robust development of biosensor using PCB substrates is mandatory for a successful future of lab-on-PCB devices. In this respect, the work reported in [11] provide a very good guide for fabricating PCB-based electrochemical biosensors. The authors analyzed the critical technological considerations, allowing the use of Printed Circuit Boards as reliable electrochemical sensing platforms. Both electrochemical and physical characterization showed that organic and inorganic sensing electrode surface includes contaminants which can be removed using several pre-cleaning techniques. In this respect, the authors proposed pre-treatment rules to fabricate PCB-based electrodes for electrochemical biosensors. They demonstrated the applicability of that methodology both for labelled protein and label-free nucleic acid biomarker quantification.

As previously commented, lab-on-PCB devices have very interesting characteristics for the biomedical applications. In particular, one of the most important application is the DNA amplification [12,13,14]. The device reported in [15] developed a lab-on-PCB for isothermal recombinase polymerase amplification (RPA) of E. coli gDNA using a commercially available 4-layer Printed Circuit Board as substrate. The device includes an integrated microheater fabricated using the copper layer of the substrate. In addition, the microheater is used as temperature sensor. The device also include a copper plate for temperature uniformity. The authors demonstrated the amplification using electrophoresis. The microfluidic chip is intended to be integrated with biosensors in a PCB substrate for the development of inexpensive point-of-care molecular diagnostics platforms.

The previously commented work performed the electrophoresis using an external device. In this respect, the device reported in [16] allows to perform the electrophoresis process using a lab on PCB. The device includes a conductivity sensor to detect the filling with Tris-acetate-EDTA (TAE) buffer. Once the TAE is detected, the device starts a controlled heating process using an integrated microheated and a SMD resistor. In addition, the device includes an optical control of the agarose preparation to finish the process. In order to do so, the authors used a Light-Dependent-Resistor (LDR) integrated in the system. Finally, the PCB substrate includes two electrodes for performing the electrophoresis. The complete system can be considered semi-automatic. However, several parts are completely automatic. This lab-on-PCB device for electrophoresis is intended to be integrated with a lab-on-PCB thermocycler to develop the complete biological process in a lab-on-PCB system.

Related to PCB-based electrophoresis applications, the device reported in [17] describes a novel planar grounded capacitively coupled contactless conductivity detector (PG-C4D) for microchip electrophoresis. The system was composed of a PCB substrate with electrodes and a poly(methyl methacrylate) (PMMA) microfluidic circuit. The reported device had lower stray capacitance than traditional capacitively coupled contactless conductivity detectors. Therefore, the baseline intensity and noise amplitude of the detection cell were smaller. This characteristic implies a higher detection sensitivity, signal-to-noise ratio, and repeatability. This proposed PCB-based PG-C4D device shows an interesting potential for electrophoresis, including, among others, the detection of industrial wastewater or environmental conditions.

An important topic of the microfluidic devices for biomedical applications, and specially for lab-on-PCB devices is the microfluidic handling of small volumes of fluids [18]. The need of microheating, sensing, and micromixing, in different parts of the PCB-based platforms makes the control of liquids necessary. The Special Issue on lab-on-PCB devices includes a review paper which describes the active fluid manipulation methods for lab-on-PCB devices, mentioning their main characteristics from the market point of view [19]. Among others, the author describes the external impulsion devices (syringe or peristaltic pumps); pressurized chambers for displacement of liquid samples and reagents; EWOD; and electro-osmotic and phase-change-based flow driving. This review is an attractive summary for researchers to chose an appropriate fluid manipulation method if they decide to use Printed Circuit Board substrates.

The potential of the lab-on-PCB devices lies on the characteristics of the different layers which compose the Printed Circuit Board substrates. In this respect, the review paper [20] describes the fabrication opportunities that Printed Circuit Boards offer for electronic and biomedical engineering. The authors comment the alternative uses of copper, gold, silver, and Flame Retardant-4 layers. In addition, they mention the use of vias, solder masks and both rigid and flexible substrates. These characteristics have been used to develop sensors, biosensors and actuators, and specially for PCB-based microfluidic platforms. The development of lab-on-PCB devices can still benefit from these alternative uses of printed circuit board layers, allowing the exploitation of commercial PCB-based biomedical and biochemical platforms.

I would like to thank all the authors for submitting their papers to the Special Issue “Lab on PCB Devices”. I also thank all the reviewers and editors for spending their time to improve the quality of the submitted papers.
